# Gestational and Early Infancy Exposure to Margarine Fortified with Vitamin D through a National Danish Programme and the Risk of Type 1 Diabetes: The D-Tect Study

**DOI:** 10.1371/journal.pone.0128631

**Published:** 2015-06-01

**Authors:** Ramune Jacobsen, Elina Hypponen, Thorkild I. A. Sørensen, Allan A. Vaag, Berit L. Heitmann

**Affiliations:** 1 Research Unit for Dietary Studies, The Parker Institute, Bispebjerg and Frederiksberg Hospitals, The Capital Region, Frederiksberg, Denmark; 2 School of Population Health, University of South Australia, Adelaide, Australia; 3 Institute of Preventive Medicine, Bispebjerg and Frederiksberg Hospitals, The Capital Region, Frederiksberg, Denmark; 4 Novo Nordisk Foundation Center for Basic Metabolic Research, University of Copenhagen, Copenhagen, Denmark; 5 MRC Integrative Epidemiology Unit, University of Bristol, Bristol, United Kingdom; 6 Department of Endocrinology, Rigshospitalet, Copenhagen, Denmark; 7 Department of Clinical Medicine, University of Copenhagen, Copenhagen, Denmark; 8 National Institute of Public Health, University of Southern Denmark, Copenhagen, Denmark; 9 Boden Institute of Obesity, Nutrition, Exercise & Eating Disorders, University of Sydney, Sydney, Australia; Faculté de médecine de Nantes, FRANCE

## Abstract

The objective of the study was to assess whether gestational and early infancy exposure to low dose vitamin D from a mandatory margarine fortification programme in Denmark influenced the risk of developing type 1 diabetes (T1D) before age of 15 years. The study population included all individuals born in Denmark from 1983 to 1988 and consisted of 331,623 individuals. The 1^st^ of June 1985, which was the date of issue of the new ministerial order cancelling mandatory fortification of margarine with vitamin D in Denmark, served as a reference point separating the studied population into various exposure groups. We further modelled birth cohort effects in children developing T1D as a linear spline, and compared the slopes between the birth cohorts with various prenatal and infancy exposures to vitamin D fortification. In total, 886 (0.26%) individuals developed T1D before the age of 15 years. The beta coefficients (95% CI), or slopes, for linear birth cohort effect in log Hazard Ratio (HR) per one month of birth in individuals born during the periods of gestational exposure, wash-out, and non-exposure were: 0.010 (-0.002/0.021), -0.010 (-0.035/0.018), and 0.008 (- 0.017/0.032), respectively. The beta coefficients (95% CI) for individuals born during the periods of first postnatal year exposure, wash-out, and non-exposure were: 0.007 (-0.016/0.030), 0.006 (-0.004/0.016), and 0.007 (-0.002/0.016), respectively. In conclusion, we found no evidence to support that exposure to low dose vitamin D from the Danish mandatory margarine fortification regimen during gestational and first postnatal year of life changed the risk of developing T1D before the age of 15 years.

## Introduction

Type 1 diabetes (T1D) is one of the rare chronic diseases starting in childhood [1]. Due to an increase in incidence, a decrease in the age of onset, and an increased survival of patients with the disease, the global prevalence of T1D has been increasing over the past decades. The T1D incidence in Danish children 0–15 years age has increased by 1.2% yearly from 1970 to 2000, and by 3.43% yearly from 1996 to 2006, with the highest incidence in the oldest age group (from 10 to 15 years), the steepest increase in incidence in the youngest age group (from 0 to 4 years), and a significant birth cohort effect among children born after the beginning of the 1980’s where the T1D incidence has been increasing linearly [2,3].

The aetiology of T1D is not fully clarified but, generally, there is no doubt that both genetic and environmental factors contribute. However, none of the presently known T1D susceptibility genes are required, or are sufficient, for overt T1D to develop [4]. A variety of environmental T1D risk and protective factors, including vitamin D supplementation during gestation and in early infancy, have been investigated [5–9].

There are several reasons to suggest that vitamin D supplementation early in life may contribute to preventing T1D. One hypothesis is that vitamin D in pregnant women is involved in the processes of immunological adaptation down-regulating the T helper type 1 (Th1) pro-inflammatory cytokine responses, that possibly contribute to the destruction of beta-cells[10]. Epigenetic changes due to environmental exposures during sensitive periods of early development—for immune priming starting in the first gestational trimester and continuing during the first years of postnatal life—may also contribute [11,12]. Animal studies demonstrated that in mice, prenatal vitamin D supplementation prevented the development of T1D [13,14]. Several epidemiological studies also found that the risk of T1D was decreased in individuals supplemented with vitamin D during gestation or infancy, and that the protective effect was directly dependent on the supplementation dose: the larger the dose, the smaller T1D risk [15].

Vitamin D is synthesized in the body from exposure to sun radiation, but animal (e.g. oily fish, eggs), and some non-animal food products (e.g. mushrooms) provide additional sources of the vitamin [16]. Oral intake might be further augmented by fortification and supplementation, which is especially important in order to maintain baseline vitamin stores during winter in populations at Northern latitudes, where the sun can initiate dermal synthesis of vitamin D only from April to October [17, 18]. A recent Danish fortification trial has proved that the intake of fortified food prevented vitamin D deficiency during winter [19].

In Denmark, it was mandatory to add vitamin D to all margarine products in a distinct time period: from January1961 to June 1985. The amount of vitamin D added to margarine was low (1.25 μg per 100 g of margarine). Still, on average, 13% of all dietary vitamin D among adult Danes was estimated to have come from the fortified margarine [20]. The reported reason to initiate mandatory fortification was the need to substitute the lack of vitamin D in margarine compared to butter, while the decision to cancel mandatory margarine fortification was justified by the low vitamin D doses added to the margarine products—an argument which, however, did not seem to be evidence-based.

The aim of this study was to assess whether gestational and early infancy exposure to small extra doses of vitamin D coming from fortified margarine influenced the risk of developing T1D later in life. We hypothesised that T1D risk will be lower among individuals born in Denmark during the period of obligatory margarine fortification—and therefore exposed to vitamin D fortified food during gestation or infancy—compared to individuals born in years when margarine was not fortified—and consequently unexposed to extra vitamin D coming from fortified margarine at any period of early life.

## Materials and Methods

### Analysis approach

**Fig 1:**
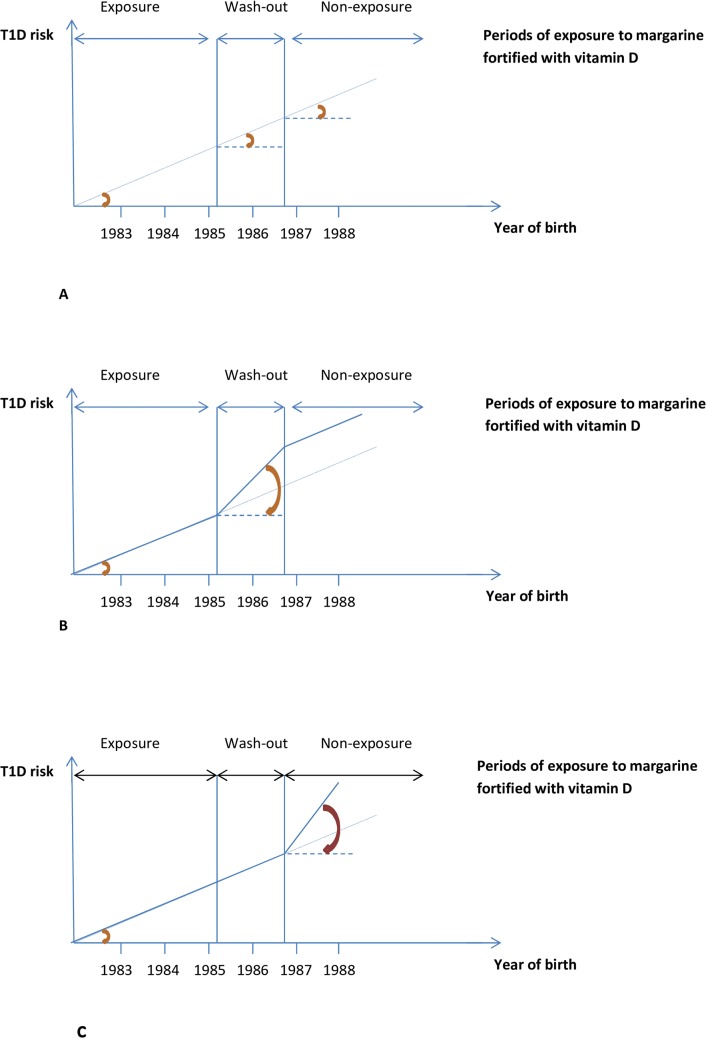
Analysis approach. A: No changes in the slope of the T1D risk trend, and no differences between the slopes in the exposure, washout and non-exposure periods. B: Increased slope during the wash-out compared to exposure period, resulting in a jump in the slope in the non-exposure compared to exposure period. C: Increase in the slope in the non-exposure compared to wash-out period resulting in a steeper slope during the non-exposure compared to exposure period.

Due to the specifics of the data (please see below under “Study population and data sources”) we analysed the exposed and unexposed to vitamin D fortification birth cohorts only around the cancellation of margarine fortification regimen. Specifically, we analysed the birth cohort effect for Danish children developing T1D up to age of 15 years and born after the beginning of the 1980’s. The 1^st^ of June 1985—date of issue of the new ministerial order on margarine cancelling obligatory fortification of margarine with vitamin D in Denmark—served as a reference point separating the studied population into exposure, washout and non-exposure groups. We modelled the birth cohort effect as a linear spline with cut-off points at the end of fortification and the end of the wash-out period, and then investigated the changes in the slopes at the cut-of points, and compared the slopes between the cohorts with various exposures. No changes (i.e. similar slopes in the exposure, wash-out, and non-exposure periods) would indicate that the T1D risk has risen steadily and exposure to fortification did not change it (Fig 1A). Higher slope during the washout period, resulting in a jump of linear T1D increase in the non-exposure compared to the exposure period (Fig 1B), and/or steeper slope in the non-exposure compared to exposure period (Fig 1C) would indicate changes in T1D risk attributable to changes in vitamin D fortification. Specifically, we expected a jump (Fig 1B) if the exposure vs. non-exposure to fortification mattered, and a slope change (Fig 1C) if an interaction between fortification and the other factors inducing the increase in incidence occurred; in other words, if the effects of these latter factors were modified by exposure to fortification. Both a jump and slope change would confirm our hypothesis that T1D risk was lower in individuals born during the period of obligatory margarine fortification, compared to individuals born after the mandatory fortification was cancelled.

### Study population and data sources

The study population was selected from the Danish Civil Registration System (CVRS), which was established in 1968, and registers all persons in Denmark alive April 1, 1968 and born thereafter [21]. In Denmark, for research purposes, the unique personal identification (CPR) numbers from the CVRS can be used for linkage with individual information from a range of other routine administrative registers and large clinical databases. From the CVRS, we received information on CPR numbers, sex, date and place of birth, and status (i.e. alive, dead, emigrated or immigrated) of all individuals born in Denmark in 1983–1988. The data were received in October 2012.

The information on the presence of T1D diagnosis and its date of occurrence (here defined as date of the first insulin injection) was obtained through the CPR number linkage with the Danish Childhood Diabetes Registry (DSDB). In Denmark, since January 1 1996, all hospitalised incident cases of T1D in children aged 0–14 years have been reported to the DSBD, where for all cases reported, the original hospital records were checked to establish the diagnosis date[3]. Thus, the DSBD contains information on all childhood diabetes cases in Denmark among individuals born since 1981 and alive or residing in the country at January 1, 1996, as well as those diagnosed after this date.

From the Danish Meteorological Institute (DMI) we obtained the values of sunshine hours in Copenhagen each month during the period 1982–1989, and used these figures to calculate cumulative gestational and first-postnatal-year sunshine hours. This was done by summing the recorded monthly sunshine hours for each individual during the full nine months prior to the month of birth, and during the full 12 months starting from the month of birth, respectively.

### Exposure periods

Taking into account that the new ministerial order cancelling mandatory margarine fortification in Denmark was introduced June 1, 1985, and (a) full nine months of gestation, (b) full four months of margarine shelf-life and (c) additional full two months of total fortified margarine washout from the households, the following birth periods for gestational exposure were developed:

- born from January 1, 1983 to May 31, 1985 formed the exposure group;
- born from June 1, 1985 to August 31, 1986 formed the washout group, and
- born from September 1, 1986 to December 31,1988 formed the non-exposure period group

The first-postnatal-year exposure groups were constructed by moving the exposure period one year backwards and were the following:

- born from January 1, 1983 to May 31, 1984 formed the exposure group;
- born from June 1, 1984 to August 31, 1986 formed the washout group, and
- born from September 1, 1986 to December 31, 1988 formed the non-exposure period group

### Statistical methods

In order to investigate changes in slopes of the linear birth cohort effect, we generated a numerical variable starting at one and lasting until 72, which indicated month and year of birth from January 1983 through December 1988. We then derived beta coefficients (or slopes) for this numerical variable in Cox regression models with two cut-off points, as described above. Age at T1D diagnosis, death, emigration, or end of follow-up (administrative censoring) served as dependent variables. We ran the separate models for gestational and first postnatal year exposures, as well as for children diagnosed up to 15 years, and at 0–4, 5–9, and 10–15 year age-periods. All models were adjusted for sex. To investigate the need for differentiated analyses among males and females, we checked the significance of formal interactions between sex and exposure periods, as well as between sex and secular trend, using likelihood ratio tests. Further, the gestational and first-postnatal-year exposure models were also run with additional adjustment for cumulative gestational and first-postnatal-year sunshine hours, respectively.

### Ethics Statement

All administrative and disease databases are accessible for research purposes in Denmark in accordance with the Danish law. For this study, the Danish Data Protection Agency provided permission to access the Danish Civil Registration System (CVR), where individual civil registration (CPR) numbers are stored (J. no.: 2012-41-41156). This permission also included permission to merge CPR numbers with different nationwide disease registers. The Steering Committee of the Danish Childhood Diabetes Registry (DSBD) gave permission to use their data. Following the permission’s requirements, the CPR numbers were immediately replaced by anonymous identification numbers after the linkage, before further handling and analyses were undertaken.

## Results

The population included 331,623 individuals born in Denmark from 1983 to 1988; 886 (0.26%) developed T1D by the age of 15 years, where the majority (503, or 56.8% of all T1D cases) were diagnosed at age 10–15 years. There were more males in the entire population (170,431 or 51.4%) as well as among those with T1D (480 or 54.2%). The size of the study population in various gestational period groups is presented in Tables 1 and 2.

**Table 1:** Studied population by periods of various gestational exposures to vitamin D fortification, T1D onset age, and sex.

N (%)	Birth period in relation to exposure to vitamin D fortification during gestation		
	Exposure: Jan 1983-May 1985*	Washout: Jun 1985-Aug 1986*	Non-exposure: Sep 1986-Dec 1988*
Total	127,207	69,667	134,749
Males (% from total)	65,266 (51.3)	35,729 (51.3)	69,436 (51.5)
T1D until 15 (% from total)	298 (0.23)	184 (0.26)	404 (0.30)
In males (% from above)	157 (52.7)	99 (53.8)	224 (55.4)
T1D at 0–4 (% from total)	32 (0.02)	28 (0.04)	54 (0.04)
In males (% from above)	21 (65.6)	11 (39.3)	35 (64.8)
T1D at 5–9 (% from total)	83 (0.07)	55 (0.08)	131 (0.10)
In males (% from above)	38 (45.8)	29 (52.7)	75 (57.3)
T1D at 10–15 (% from total)	183 (0.14)	101 (0.14)	219 (0.16)
In males (% from above)	98 (53.6)	59 (58.4)	114 (52.1)

* including the starting and ending months.

**Table 2:** Studied population by periods of various first postnatal year exposures to vitamin D fortification, T1D onset age, and sex.

N (%)	Birth period in relation to exposure to vitamin D fortification during first year of postnatal life		
	Exposure: Jan 1983-May 1984*	Washout: Jun 1984-Aug 1986*	Non-exposure: Sep 1986-Dec 1988*
Total	73,986	122,888	134,749
Males (% from total)	37,977 (51.3)	63,018 (51.3)	69,436 (51.5)
T1D until 15 (% from total)	156 (0.23)	326 (0.26)	404 (0.30)
In males (% from above)	82 (52.6)	174 (53.4)	224 (55.4)
T1D at 0–4 (% from total)	15 (0.02)	45 (0.04)	54 (0.04)
In males (% from above)	7 (46.7)	25 (55.6)	35 (64.8)
T1D at 5–9 (% from total)	49 (0.07)	89 (0.08)	131 (0.10)
In males (% from above)	19 (38.8)	48 (53.9)	75 (57.3)
T1D at 10–15 (% from total)	92 (0.14)	192 (0.14)	219 (0.16)
In males (% from above)	56 (60.9)	101 (52.6)	114 (52.1)

* including the starting and ending months.

**Fig 2:**
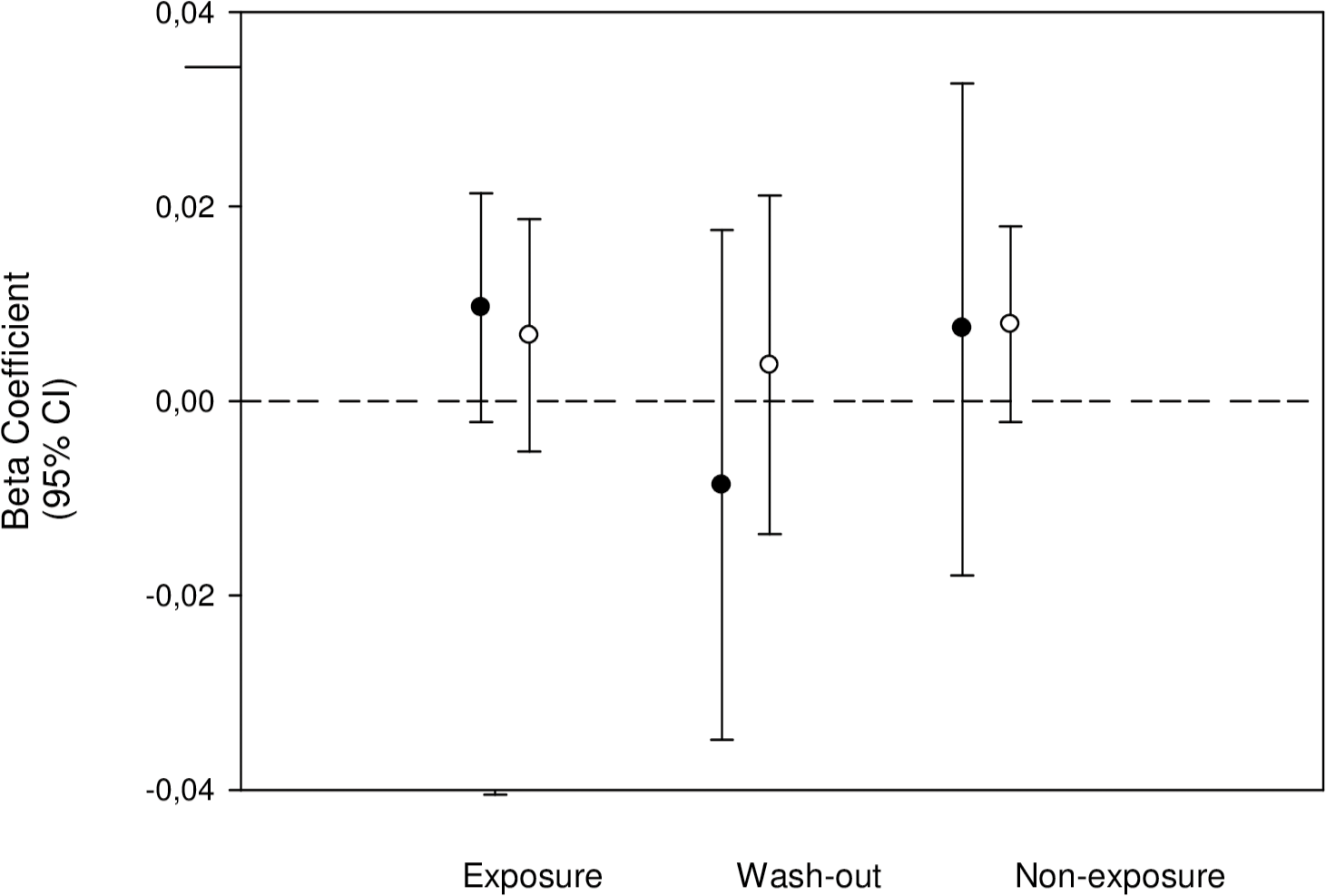
The slopes (95% CI) for linear change in T1D risk among individuals with various gestational exposures to margarine fortification. The slopes, or beta regression coefficients, are expressed in log HR per month of birth; in black—adjusted for sex, in white—adjusted for sex and cumulative gestational sunshine.

**Fig 3:**
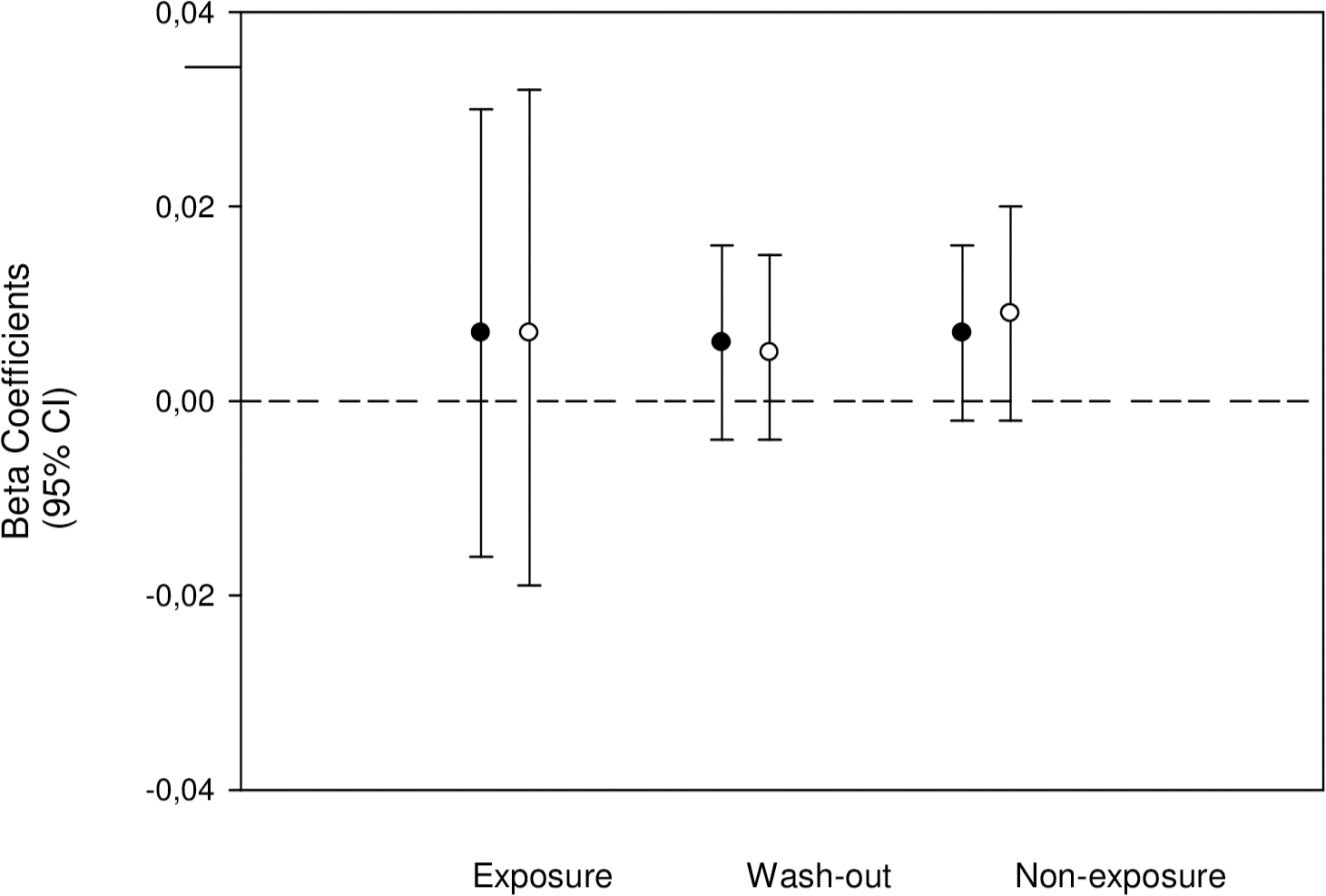
Slopes (95% CI) for linear change in T1D risk among individuals with various first postnatal year exposures to margarine fortification. The slopes, or beta regression coefficients, are expressed in log HR per month of birth; in black – adjusted for sex, in white – adjusted for sex and cumulative gestational sunshine.

The beta coefficients (95% CI), or slopes, for linear increase in the risk of developing T1D until age 15 years, after adjustment for sex, was 0.007 (0.003/0.0,010), p<0.001, log Hazard Ratio (HR) per one month of birth. The slopes did not differ between individuals from the various gestational or first-year postnatal exposure groups, suggesting that the T1D risk has risen steadily, and that exposure to the margarine fortification did not change the pattern (Fig 1A); results were essentially similar after the adjustment for cumulative sunshine hours during the respective exposure periods (Figs 2 and 3). The results were also similar for all age groups and for both types of exposures (S1 and S2 Tables). There were no significant interactions between sex and secular trend, or sex and any type of exposure (p> 0.05 for all comparisons), suggesting that sex did not modify the slope pattern and that the differentiated analyses for males and females were not needed.

## Discussion

Mandatory fortification of margarine with vitamin D has been applied during various time periods since the 1930’s in the Scandinavian countries [20]. The present study assesses the health benefits of fortification in Denmark. When assessing the benefits of food fortification, mostly medium scale clinical trials are conducted, so far showing that vitamin D-fortified foods improve vitamin D status in adults [22]. However, fortification trials do not usually have the power or design to assess fortification effects on health outcomes. Additionally, in Finland, where vitamin D since February 2003 has been added to all liquid milk products, margarine and butter, before-after comparisons in various cohorts showed that fortification has improved the vitamin D status in both children and adults [23,24]. On the background of the great need of vitamin D in Finland due to its high latitude, this could have contributed to the recent levelling-off of the otherwise very high T1D incidence in 0–15 year old children [25]. In the present study, we investigated early life exposure to fortification, and its association with the later T1D risk, and utilized the fact that the mandatory Danish vitamin D fortification regimen was abandoned at a distinct time point. Our study thus resembled studies exploring early origins of diseases by using evidence from natural or societal experiments, such as famines—an approach that clearly is suitable for food fortification assessment studies.

This study confirmed the linear increasing trend in the incidence of childhood T1D in individuals born in Denmark from 1983 to 1988, and we found no evidence to support that the early life exposure to small extra doses of vitamin D from the Danish mandatory margarine fortification programme affected this trend. One reason for the absence of a detectable relationship between TD risk and early life exposure to fortified margarine could be the actual dose of vitamin D added to margarine, and therefore the dose which individuals were exposed to during gestation and/or during the first year of life. The amount of vitamin D added to margarine was 1.25 μg per 100g [20]. According to results from previous studies, pregnant Danish women are getting approximately 3 μg of vitamin D per day from the diet [26], which in turn suggests that the amount of vitamin D coming from the fortified margarine (i.e. 13%) may have been approximately 0.40 μg per day. This figure agrees well with information from food disappearance data showing that Danish citizens, during the period between 1983 and 1988, were buying on average 16,000–17,000 g margarine per person per year (equal to 44–46 g per person per day), and thus taking on average 0.55–0.57 μg of vitamin D per person day from the fortified margarine [27]. The intake of vitamin D among pregnant and lactating women correlates well with the vitamin D serum levels [28]. Because vitamin D travels freely through the placenta, the doses of foetal vitamin D will be similar with the doses that the pregnant women were getting [29]. On the other hand, only a small proportion of vitamin D passes from the maternal circulation to breast milk[30]. As the maternal vitamin D fortification had effect on babies´ vitamin D levels via breast feeding, vitamin D intake during the first year of life was likely to have been lower than the maternal intake. Additionally, some babies could have been getting small doses of vitamin D via the complementary feeding prepared using the fortified margarine. Still, the infants’ vitamin D intake from the fortified margarine could not be higher than 0.40–0.55 μg per person per day, as calculated above. In comparison, in the previously conducted Finnish cohort study, which showed a decrease in T1D of around 20%, the vitamin D supplementation doses given to infants were 50 μg per day [31].

Another potential reason why we did not detect effects of early exposure to vitamin D fortification on later T1D risk may be related to statistical power issues [32]. Although increasing, the incidence of T1D in absolute numbers was low, and the Danish national sample including birth cohorts from two calendar years was not sufficiently large to detect the effect of exposure to fortification on T1D risk. On the other hand, expanding the sample to include more birth years, and hence individuals born prior to and after the fortification policy shift in June 1985, would introduce confounding and bias because of the greater age, period and cohort differences potentially introduced.

A strong feature of our study set up was that the individuals included in the analyses were from adjacent birth cohorts, and that they were unselected with respect to mother’s age, individual diet intake, use of supplements, breast feeding length, delivery type, gestational age at birth, and birth order. All of these factors, which in different ways may be related to either vitamin D intake and/or T1D risk, can be assumed to be balanced between the individuals from the various exposure groups, and therefore confounding is not to be expected. However, we needed to consider other potential societal events occurring simultaneously with the changes in fortification practice, as well as confounders changing over time during 1983–1988.We are unaware of any significant relevant societal changes occurring simultaneously in time with the mandatory margarine fortification cancellation. Among the potential confounders changing over time, we explored the changes in Danes’ margarine consumption. According to Danish food disappearance statistics [27], margarine intake during the period from 1983 to 1985 decreased only slightly, and these changes were most likely too small to be expected to influence the interpretation of the current results.

Another strong feature of the study was the use of DSDB to identify individuals with T1D diagnoses. The registry includes only verified cases of T1D diagnosed up to the age of 15 years, and has been proved to be valid and reliable [3].

One limitation of the study was that a similar trend analysis around the initiation of the margarine fortification programme in 1961 could not be done, as we lack complete and valid information on T1D diagnoses before the age of 15 years. Additionally, those unexposed around the initiation would have presented individuals unexposed during gestation or first year of life, and also unexposed during mother’s pre-pregnancy. When only analysing the period around the cessation of the fortification in 1985, we may have had the problem of pre-pregnancy loading which potentially could matter as much as exposure during gestation and during infancy.

## Conclusions

We did not find that gestational or early infancy exposure to small extra doses of vitamin D coming from the Danish mandatory margarine fortification programme influenced the risk of developing T1D later in life.

## Supporting Information

S1 Table Slopes (95% CI) for linear increase in T1D incidence for individuals born during various periods of gestational exposure to vitamin D fortification by age at T1D diagnosis. The slopes, or regression coefficients, are expressed in log HR per month of birth; all adjusted for sex, in italic—adjusted for sex and cumulative gestational sunshine. ^1^ Administratively censored at age of 5; ^2^ administratively censored at age of 10 and truncated before age of 5; ^3^ truncated at before age of 10; ^4^ including the starting and ending months.
(DOCX)

S2 Table Slopes (95% CI) for linear increase in T1D incidence for individuals during various periods of first postnatal year exposure to vitamin D fortification by age at T1D diagnosis. The slopes, or regression coefficient, are expressed in log HR per month of birth; all adjusted for sex, in italic—adjusted for sex and cumulative 1^st^ postnatal year sunshine. ^1^ Administratively censored at age of 5; ^2^ administratively censored at age of 10 and truncated before age of 5; ^3^ truncated before age of 10; ^4^ including the starting and ending months.
(DOCX)

## References

[1] Diagnosis and classification of diabetes mellitus. Diabetes Care. 2008; 31 Suppl 1: S55–S60. 10.2337/dc08-S055 18165338

[2] Svensson J, Carstensen B, Molbak A, Christau B, Mortensen HB, Nerup J et al Increased risk of childhood type 1 diabetes in children born after 1985. Diabetes Care. 2002; 25: 2197–2201. 12453960 10.2337/diacare.25.12.2197

[3] Svensson J, Lyngaae-Jorgensen A, Carstensen B, Simonsen LB, Mortensen HB. Long-term trends in the incidence of type 1 diabetes in Denmark: the seasonal variation changes over time. Pediatr Diabetes. 2009; 10: 248–254. 10.1111/j.1399-5448.2008.00483.x 19067889

[4] Atkinson MA, Eisenbarth GS, Michels AW. Type 1 diabetes. Lancet. 2014; 383: 69–82. 10.1016/S0140-6736(13)60591-7 23890997 PMC4380133

[5] Cardwell CR, Carson DJ, Patterson CC. Parental age at delivery, birth order, birth weight and gestational age are associated with the risk of childhood Type 1 diabetes: a UK regional retrospective cohort study. Diabet Med. 2005; 22: 200–206. 15660739 10.1111/j.1464-5491.2005.01369.x

[6] Cardwell CR, Stene LC, Joner G, Cinek O, Svensson J, Goldacre MJ et al Caesarean section is associated with an increased risk of childhood-onset type 1 diabetes mellitus: a meta-analysis of observational studies. Diabetologia. 2008; 51: 726–735. 10.1007/s00125-008-0941-z 18292986

[7] Cardwell CR, Carson DJ, Patterson CC. No association between routinely recorded infections in early life and subsequent risk of childhood-onset Type 1 diabetes: a matched case-control study using the UK General Practice Research Database. Diabet Med. 2008; 25: 261–267. 10.1111/j.1464-5491.2007.02351.x 18201209

[8] Cardwell CR, Stene LC, Joner G, Davis EA, Cinek O, Rosenbauer J et al Birthweight and the risk of childhood-onset type 1 diabetes: a meta-analysis of observational studies using individual patient data. Diabetologia. 2010; 53: 641–651. 10.1007/s00125-009-1648-5 20063147

[9] Svensson J, Carstensen B, Mortensen HB, Borch-Johnsen K. Early childhood risk factors associated with type 1 diabetes—is gender important? Eur J Epidemiol. 2005; 20: 429–434. 16080591 10.1007/s10654-005-0878-1

[10] Hypponen E. Preventing vitamin D deficiency in pregnancy: importance for the mother and child. Ann Nutr Metab. 2011; 59: 28–31. 10.1159/000334150 22123634

[11] Barker DJ. In utero programming of chronic disease. Clin Sci (Lond). 1998; 95: 115–128. 9680492

[12] Gluckman PD, Hanson MA, Cooper C, Thornburg KL. Effect of in utero and early-life conditions on adult health and disease. N Engl J Med. 2008; 359: 61–73. 10.1056/NEJMra0708473 18596274 PMC3923653

[13] Giulietti A, Gysemans C, Stoffels K, van EE, Decallonne B, Overbergh L et al Vitamin D deficiency in early life accelerates Type 1 diabetes in non-obese diabetic mice. Diabetologia. 2004; 47: 451–462. 14758446 10.1007/s00125-004-1329-3

[14] Mathieu C, Waer M, Laureys J, Rutgeerts O, Bouillon R. Prevention of autoimmune diabetes in NOD mice by 1,25 dihydroxyvitamin D3. Diabetologia. 1994; 37: 552–558. 7926338 10.1007/BF00403372

[15] Zipitis CS, Akobeng AK. Vitamin D supplementation in early childhood and risk of type 1 diabetes: a systematic review and meta-analysis. Arch Dis Child. 2008; 93: 512–517. 10.1136/adc.2007.128579 18339654

[16] Lips P. Vitamin D physiology. Prog Biophys Mol Biol. 2006; 92: 4–8. 16563471 10.1016/j.pbiomolbio.2006.02.016

[17] Brot C, Vestergaard P, Kolthoff N, Gram J, Hermann AP, Hermann AP et al Vitamin D status and its adequacy in healthy Danish perimenopausal women: relationships to dietary intake, sun exposure and serum parathyroid hormone. Br J Nutr. 2001; 86 Suppl 1: S97–103. 11520426 10.1079/bjn2001345

[18] Mejborn H, Brot C, Hansen HB, Koch B, Hyldstrup L, Mortensen L et al. D-vitaminstatus i den danske befolkning bør forbedres (Vitamin D status in Danish population should be improved). Copenhagen: The Ministry of Food, Agriculture and Fisheries of Denmark; 2004

[19] Madsen KH, Rasmussen LB, Andersen R, Molgaard C, Jakobsen J, Bjerrum PJ et al Randomized controlled trial of the effects of vitamin D-fortified milk and bread on serum 25-hydroxyvitamin D concentrations in families in Denmark during winter: the VitmaD study. Am J Clin Nutr. 2013; 98: 374–382. 10.3945/ajcn.113.059469 23783292

[20] Haradsdóttir J. and Thaarup S. Tilsætning af vitaminer og minraler til levnedsmidler (The fortification of foods with vitamins and minerals) Copenhagen: Nordic Council of Ministers; 1989.

[21] Pedersen CB. The Danish Civil Registration System. Scan J Pub Health. 2011; 39: 22–25. 10.1177/1403494810387965 21775345

[22] Black LJ, Seamans KM, Cashman KD, Kiely M. An updated systematic review and meta-analysis of the efficacy of vitamin D food fortification. J Nutr. 2012; 142: 1102–1108. 10.3945/jn.112.158014 22513988

[23] Laaksi IT, Ruohola JP, Ylikomi TJ, Auvinen A, Haataja RI, Pihlajamaki HK et al Vitamin D fortification as public health policy: significant improvement in vitamin D status in young Finnish men. Eur J Clin Nutr. 2006; 60: 1035–1038. 16482069 10.1038/sj.ejcn.1602414

[24] Piirainen T, Laitinen K, Isolauri E. Impact of national fortification of fluid milks and margarines with vitamin D on dietary intake and serum 25-hydroxyvitamin D concentration in 4-year-old children. Eur J Clin Nutr. 2007; 61: 123–128. 16885927 10.1038/sj.ejcn.1602506

[25] Harjutsalo V, Sund R, Knip M, Groop PH. Incidence of type 1 diabetes in Finland. JAMA. 2013; 310: 427–428. 10.1001/jama.2013.8399 23917294

[26] Jensen CB, Petersen SB, Granstrom C, Maslova E, Molgaard C, Olsen SF. Sources and determinants of vitamin D intake in Danish pregnant women. Nutrients. 2012; 4: 259–272. 10.3390/nu4040259 22606369 PMC3347007

[27] Fagt S and Trolle E. Forsyningen af fødevarer 1955–1999. Udviklingen i danskernes kost—forbrug, indkøb og vaner (The supply of food from 1955 to 1999. Overview of the developments in the Danish diet—consumption, purchasing and habits). Søborg: Danish Directorate for Food Business; 2001.

[28] Hollis BW, Wagner CL. Nutritional vitamin D status during pregnancy: reasons for concern. CMAJ. 2006; 174: 1287–1290. 16636329 10.1503/cmaj.060149 PMC1435950

[29] Bowyer L, Catling-Paull C, Diamond T, Homer C, Davis G, Craig MJ. Vitamin D, PTH and calcium levels in pregnant women and their neonates. Clin Endocrinol (Oxf). 2009; 70: 372–377. 10.1111/j.1365-2265.2008.03316.x 18573121

[30] Thiele DK, Senti JL, Anderson CM. Maternal vitamin D supplementation to meet the needs of the breastfed infant: a systematic review. J Hum Lact. 2013; 29: 163–170. 10.1177/0890334413477916 23458952

[31] Hypponen E, Laara E, Reunanen A, Jarvelin MR, Virtanen SM. Intake of vitamin D and risk of type 1 diabetes: a birth-cohort study. Lancet. 2001; 358: 1500–1503. 11705562 10.1016/S0140-6736(01)06580-1

[32] Altman DG, Bland JM. Absence of evidence is not evidence of absence. Aust Vet J. 1996; 74: 311 8937675 10.1111/j.1751-0813.1996.tb13786.x

