# Supplementary material for: Gestational and Early Infancy Exposure to Margarine Fortified with Vitamin D through a National Danish Programme and the Risk of Type 1 Diabetes: The D-Tect Study
Source: PLoS One. 2015 Jun 1;10(6):e0128631. doi: 10.1371/journal.pone.0128631 (PMC4452099; doi:10.1371/journal.pone.0128631)
Supplement: S1 Table — The slopes, or regression coefficients, are expressed in log HR per month of birth; all adjusted for sex, in italic—adjusted for sex and cumulative gestational sunshine. 1 Administratively censored at age of 5; 2 administratively censored at age of 10 and truncated before age of 5; 3 truncated at before age of 10; 4 including the starting and ending months. (DOCX) [file pone.0128631.s001.docx]

**S1 Table. Slopes (95% CI) for linear increase in T1D incidence for individuals born during various periods of gestational exposure to vitamin D fortification by age at T1D diagnosis.**

| **Age of onset of T1D** | **Birth period of exposure to vitamin D fortification during gestation** | | |
| --- | --- | --- | --- |
|  | **Exposure**  **Jan 1983-May 1985⁴** | **Washout**  **Jun 1985-Aug 1986⁴** | **Non-exposure**  **Sep 1986-Dec 1988⁴** |
| **0-4¹** | 0.018 (-0.017/0.053)  *0.018 (-0.018/0.053)* | 0.023 (-0.024/0.069)  *0.023 (-0.024/0.070)* | -0.010 (-0.037/0.018)  *-0.010 (-0.037/0.018)* |
| **5-9²** | 0.006 (-0.016/0.028)  *0.000 (-0.022/0.022)* | 0.006 (-0.025/0.038)  *0.011 (-0.020/0.043)* | 0.014 (-0.004/0.032)  *0.013 (-0.005/0.031)* |
| **10-15³** | 0.011 (-0.004/0.026)  *0.010 (-0.006/0.025)* | -0.009 (-0.031/0.014)  *-0.018 (-0.052/0.016)* | 0.010 (-0.004/0.023)  *0.018 (-0.015/0.051)* |

The slopes, or regression coefficients, are expressed in log HR per month of birth; all adjusted for sex, in italic - adjusted for sex and cumulative gestational sunshine. ¹ Administratively censored at age of 5; ² administratively censored at age of 10 and truncated before age of 5 ;³ truncated at before age of 10 ; ⁴ including the starting and ending months.
