# Supplementary material for: Gestational and Early Infancy Exposure to Margarine Fortified with Vitamin D through a National Danish Programme and the Risk of Type 1 Diabetes: The D-Tect Study
Source: PLoS One. 2015 Jun 1;10(6):e0128631. doi: 10.1371/journal.pone.0128631 (PMC4452099; doi:10.1371/journal.pone.0128631)
Supplement: S2 Table — The slopes, or regression coefficient, are expressed in log HR per month of birth; all adjusted for sex, in italic—adjusted for sex and cumulative 1st postnatal year sunshine. 1 Administratively censored at age of 5; 2 administratively censored at age of 10 and truncated before age of 5; 3 truncated before age of 10; 4 including the starting and ending months. (DOCX) [file pone.0128631.s002.docx]

**S2 Table. Slopes (95% CI) for linear increase in T1D incidence for individuals during various periods of first postnatal year exposure to vitamin D fortification by age at T1D diagnosis.**

| **Age of onset of T1D** | **Birth period of exposure to vitamin D fortification during first year of life** | | |
| --- | --- | --- | --- |
|  | **Exposure**  **Jan 1983-May 1985⁴** | **Washout**  **Jun 1985-Aug 1986⁴** | **Non-exposure**  **Sep 1986-Dec 1988⁴** |
| **0-4¹** | 0.011 (-0.059/0.081)  *0.015 (-0.060/0.090)* | 0.022 (-0.004/0.049)  0.022 (-0.004/0.049) | -0.010 (-0.037/0.018)  *-0.012 (-0.041/0.017)* |
| **5-9²** | -0.004 (-0.047/0.038)  *-0.004 (-0.049/0.042)* | 0.010 (-0.008/0.027)  0.008 (-0.010/0.027) | 0.013 (-0.004/0.029)  *0.012 (-0.008/0.032)* |
| **10-15³** | 0.015 (-0.015/0.026)  *0.010 (-0.023/0.042)* | -0.001 (-0.013/0.011)  -0.001 (-0.014/0.011) | 0.008 (-0.005/0.020)  *0.011 (-0.004/0.026)* |

The slopes, or regression coefficient, are expressed in log HR per month of birth; all adjusted for sex, in italic – adjusted for sex and cumulative 1^st^ postnatal year sunshine. ¹ Administratively censored at age of 5; ² administratively censored at age of 10 and truncated before age of 5; ³ truncated before age of 10; ⁴ including the starting and ending months.
